# Onco-Multi-OMICS Approach: A New Frontier in Cancer Research

**DOI:** 10.1155/2018/9836256

**Published:** 2018-10-03

**Authors:** Sajib Chakraborty, Md. Ismail Hosen, Musaddeque Ahmed, Hossain Uddin Shekhar

**Affiliations:** ^1^Department of Biochemistry and Molecular Biology, University of Dhaka, Dhaka 1000, Bangladesh; ^2^Princess Margaret Cancer Centre/University Health Network, Toronto, Ontario, Canada

## Abstract

The acquisition of cancer hallmarks requires molecular alterations at multiple levels including genome, epigenome, transcriptome, proteome, and metabolome. In the past decade, numerous attempts have been made to untangle the molecular mechanisms of carcinogenesis involving single OMICS approaches such as scanning the genome for cancer-specific mutations and identifying altered epigenetic-landscapes within cancer cells or by exploring the differential expression of mRNA and protein through transcriptomics and proteomics techniques, respectively. While these single-level OMICS approaches have contributed towards the identification of cancer-specific mutations, epigenetic alterations, and molecular subtyping of tumors based on gene/protein-expression, they lack the resolving-power to establish the casual relationship between molecular signatures and the phenotypic manifestation of cancer hallmarks. In contrast, the multi-OMICS approaches involving the interrogation of the cancer cells/tissues in multiple dimensions have the potential to uncover the intricate molecular mechanism underlying different phenotypic manifestations of cancer hallmarks such as metastasis and angiogenesis. Moreover, multi-OMICS approaches can be used to dissect the cellular response to chemo- or immunotherapy as well as discover molecular candidates with diagnostic/prognostic value. In this review, we focused on the applications of different multi-OMICS approaches in the field of cancer research and discussed how these approaches are shaping the field of personalized oncomedicine. We have highlighted pioneering studies from “The Cancer Genome Atlas (TCGA)” consortium encompassing integrated OMICS analysis of over 11,000 tumors from 33 most prevalent forms of cancer. Accumulation of huge cancer-specific multi-OMICS data in repositories like TCGA provides a unique opportunity for the systems biology approach to tackle the complexity of cancer cells through the unification of experimental data and computational/mathematical models. In future, systems biology based approach is likely to predict the phenotypic changes of cancer cells upon chemo-/immunotherapy treatment. This review is sought to encourage investigators to bring these different approaches together for interrogating cancer at molecular, cellular, and systems levels.

## 1. Introduction to “OMICS” Technologies

“OMICS” technologies are characterized by high-throughput interfaces which facilitate the investigation of genome, epigenome, transcriptome, proteome, and metabolome in a global-unbiased manner. OMICS techniques are now being used to understand the intricate biological systems and uncover the molecular signatures underlying the complex cellular phenotypes [1, 2]. Different OMICS approaches were developed to untangle the complexity of biological systems at different dimensions (e.g., gene, RNA, and protein levels). Recent advancements of OMICS techniques have been proved to be the weapon of choice to dissect the aberrant cellular functions that lay in the heart of multifactorial diseases such as cancer [1].

### 1.1. Increase of Complexity from Genome to Proteome

**Figure 1:**
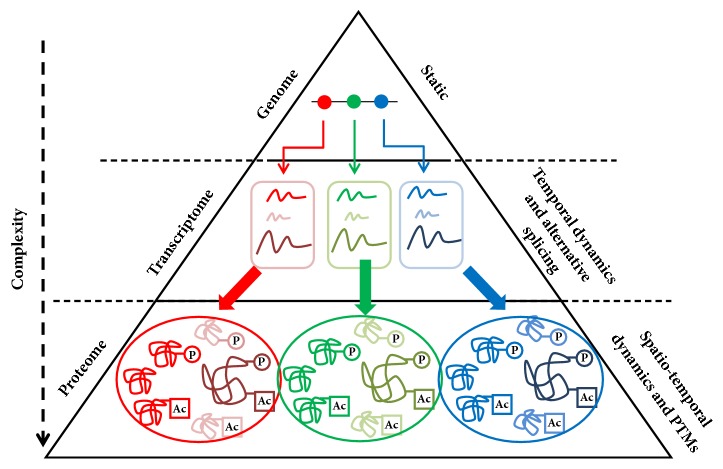
**Pyramid of complexity. **The pyramid represents the flow of information from genome (top) to transcriptome (middle), to proteome (bottom). The complexity increases from genome to proteome (indicated by down arrow). The complexity of transcriptome is largely mediated by temporal dynamics and alternative splicing. In contrast, spatiotemporal dynamics and posttranslational modifications (PTMs) are mainly responsible for high proteome complexity. Examples of PTMs include phosphorylation (P) and acetylation (Ac).

**Figure 2:**
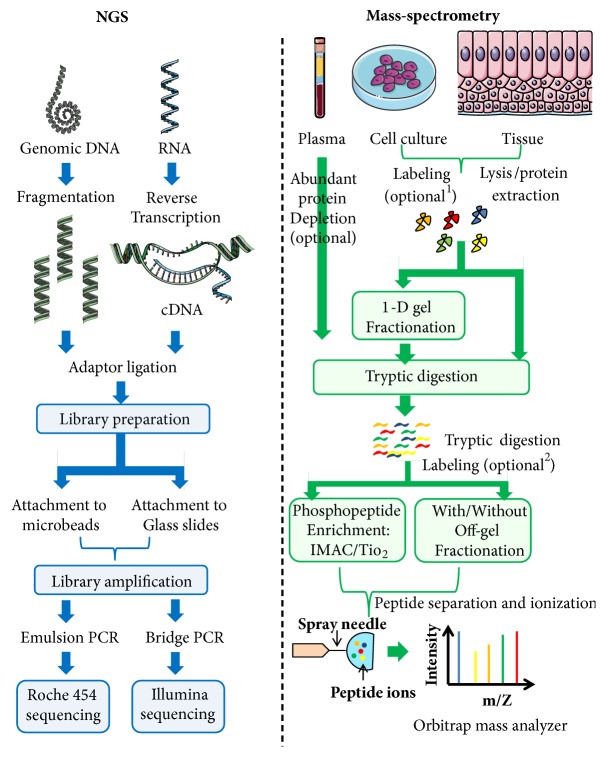
**Schematic diagram representing the basic steps of NGS and mass-spectrometry.** NGS (left) can be used for both genomic DNA and RNA-sequencing. Mass-spectrometry based proteomics (right) are typically used to identify and quantify large amount of proteins from cells, tissues, and body fluids. ^1^Primary cells and tissues from patients can be mixed with labelled proteins, typically extracted from cell lines cultured in presence of stable isotopically labelled amino acids. This method is called Super-SILAC. Proteomics can also be done without any labelling steps. This method is known as label free-quantification (LFQ). ^2^Peptides obtained after tryptic digestion can also be labelled chemically by methods known as “Tandem Mass Tag (TMT)” or “Isobaric tags for relative and absolute quantitation (iTRAQ)”.

The different OMICS levels—Genomics, Transcriptomics, and Proteomics—vary greatly in their complexity that is largely driven by the spatial- and/or temporal dynamics and chemical modifications (Figure 1). The flow of information from DNA to RNA and ultimately to protein is accompanied by an exponential increase in the complexity. The hereditary information stored in the genome in the form of 4 nucleotides remains largely static but temporal dynamics is introduced in the process of transcription by which genes are transcribed into RNAs. Orchestration of temporal regulation of gene expression depending on developmental, environmental, and extracellular cues via gene-regulatory networks makes the transcriptome a highly dynamic entity [3]. Alternative splicing in addition to temporal dynamics increases the complexity of transcriptome. mRNAs are engaged into even more complex information coding systems: translation process where mRNAs encode for proteins comprising 20 amino acids. After synthesis, proteins are typically folded into many possible conformations depending on the primary amino acid sequences and chemical modification of amino acid residues known as posttranslational modifications (PTMs). Proteins undergo a large number of PTMs (e.g., phosphorylation, acetylation, and glycosylation) that may directly affect their structure and functionality. Moreover, unlike mRNAs which are synthesized in nucleus and translated in cytoplasm, proteins have different subcellular localizations such as cell membrane, cytoplasm, and different membrane bound subcellular organelles—nucleus, mitochondria, endoplasmic reticulum, etc. Altogether these events confer huge complexity to the proteome. Two most important technologies—next-generation sequencing (NGS) and mass-spectrometry (LC-MS/MS)—have revolutionized the field of OMICS by deciphering the human genome, transcriptome, and proteome. Schematic diagram representing the typical workflow of NGS (left panel) and mass-spectrometry (right panel) experiments is shown in Figure 2.

### 1.2. Next-Generation Sequencing Based Approaches: Genomics, Epigenomics, and Transcriptomics

In recent years, the genomics-techniques are mostly dedicated to sequence the genome of an individual to understand the interindividual variations at both the germline and somatic levels. The eventual graduation of the sequencing technologies from the Sanger sequencing based approaches to the NGS-based massively parallel sequencing has enabled researchers to sequence the genome/exome of interest deeply enough to characterize the mutational landscapes of a given tissue. For example, in a large scale project known as “The Cancer Genome Atlas (TCGA)”, the scientists employed the NGS coupled with downstream bioinformatics analysis to discover somatic mutational landscape across thousands of tumor samples representing major cancer types under the assumption that these genome-wide mutational studies would be pivotal in understanding complexity of different cancer [4, 5].

Epigenomics is defined by the genome-wide identifications of chemical modifications such as methylation and acetylation of DNA and/or DNA-binding histone proteins. Epigenetic-modifications of DNA and histones proteins serve as a major regulatory mechanism controlling gene expression and cellular phenotypes [6]. The epigenomics studies have played integral role in uncovering the disease-associated epigenetic markers. Epigenomics techniques that are widely used include chromatin immunoprecipitation (ChIP) assays coupled NGS commonly known as ChIP-Sequencing or ChIP-seq and methylation analysis through whole-genome bisulfite/array-based sequencing. ChIP-Seq has been developed as a powerful tool for the identification of DNA-binding sites for transcription factors (TFs) and histone proteins in a genome-wide manner to construct high-resolution genome-wide maps of histone modification marks. ChIP-seq follows a straightforward protocol where DNA-bound proteins are typically immunoprecipitated by specific antibody followed by the extraction, purification, and sequencing of the bound DNA. In recent years application of ChIP-seq has enabled us to gain deep-insights into gene-regulatory events that are responsible for various diseases and biological pathways, such as cancer progression and development, respectively. By comparing these genome-wide profiles of histone modifications marks between cancer and normal tissues it has been possible to understand how epigenetic deregulation manifested in various cancers such as breast [7] and lung [8]. Apart from histone modifications, chemical modifications in certain DNA base can have dramatic epigenetic effects. For instance, chemical modification of Cytosine residue in the promoter DNA sequence of genes can modify their expression. By harnessing the power of NGS, it is now possible to analyze genome-wide methylome patterns at a single nucleotide resolution. Whole-genome bisulfite sequencing (WGBS) or Bisulfite sequencing (BS-Seq) in short is a powerful technology that can detect the methylated Cytosine bases in genomic DNA. In brief the method involves the treatment of genomic DNA with sodium bisulfite followed by sequencing to construct a genome-wide map of methylated Cytosine with single-base resolution. Apart from this, a relatively novel technique known as MBD-isolated Genome Sequencing (MiGS) has recently been used to analyze whole-genome methylation pattern [9]. This technique relies on the precipitation of methylated DNA by recombinant methyl-CpG binding domain of MBD2 protein followed by sequencing. A study by Vidal et al. investigated the genome-wide methylation analysis of 1112 primary tumors of various cancers types where the authors identified hypermethylated promoters and enhancers that regulate the expression of tumor-suppressor genes and concluded that changes in DNA methylation pattern tend to occur in the onset, progression, and dissemination of cancer [10].

Transcriptomics techniques are engaged in the detection of the presence and quantification of RNA transcripts especially mRNAs but can also be extended to other types of noncoding RNA transcripts such as long noncoding transcripts (LncRNAs) and microRNAs. Transcriptome in a particular cell includes all RNA molecules that are transcribed from the genome at a given time. In contrast to the genome, which is static in nature, the transcriptome is subjected to change depending on cellular, environment, extracellular, and developmental stimuli in temporal manner. Before the advent of NGS, microarray was used as the conventional laboratory technique to detect the changes in the mRNA levels within the cells at different stages in a high-throughput manner. Microarrays can typically be used to quantify the relative abundance of mRNAs for thousands of genes simultaneously. By this technique, it is possible to construct cellular or tissue gene expression profiles between normal and cancer states which may facilitate the identification of genes that exhibit differential expression between normal and cancer states.

Leveraged by the development of efficient NGS techniques, the cutting edge “Transcriptomics” technique—RNA-sequencing (RNA-seq)—can identify the presence as well as the abundance of RNA transcripts in an unbiased genome-wide manner (Figure 2). Unlike microarrays, RNA-seq technology does not rely upon the transcript-specific probes and thus can successfully perform the unbiased detection of novel transcripts. The other advantages that the RNA-seq offers over microarrays include broad dynamic range, increased specificity/sensitivity, and detection of low abundant transcripts. RNA-Seq analysis has shown that the mammalian transcriptional landscape is much more complex than was previously imagined before. Apart from diverse range of protein-coding RNAs and well established regulatory RNAs such as microRNAs, different types of noncoding RNAs (ncRNAs) are pervasively transcribed from the vast majority of noncoding regions of the genome including intergenic and intronic sequences [11]. The recent influx of huge RNA-seq data has revealed a differential gene expression pattern between various types of cancer tissues and their normal counterparts and thus harbors the potential to uncover the intricate molecular mechanisms to understand the progression of cancer [12]. The huge data repositories such TCGA offer the opportunity to reanalyze the OMICS data by a pan-cancer approach where different types of cancers can be compared and contrasted in terms of genomic and transcriptomic landscapes [13]. Li et al. comprehensively analyzed the gene expression profiles across 33 human cancer types from the TCGA database and identified up- and downregulated genes that exhibited remarkable consistency across different cancer [12].  Table 1 represents summary of the applications of different NGS-based OMICS techniques.

### 1.3. Mass-Spectrometry (LC-MS/MS) Based Techniques: Proteomics and Metabolomics

While transcriptomics is dedicated to the measurement of RNA transcripts, proteomics is specialized in the identification and quantification of the proteins that are present at a given time in biological samples. Unlike the transcriptomics, quantification of the proteome requires special strategies, since the identification and quantification of proteins in large scale are challenging due to the high complexity and dynamic range of the proteome. Transcriptomics platforms such as RNAseq-based approaches are designed to reveal the information at the transcriptome level that in turn shapes the proteome to carry out the functional cellular processes. Since most of the biological processes are controlled by proteins, it is important to reliably and accurately measure proteome alterations in aberrant cellular state such as in a cancer context to understand how cellular processes are carried out mechanistically. However, genome-wide proteomics data for cancer is exasperatingly limited. To tackle this problem as a part of TCGA a protein-expression dataset for a large number of tumor samples and cell lines has been generated using reverse-phase protein arrays (RPPAs) which is called The Cancer Proteome Atlas (TCPA)” [14]. TCPA utilized antibodies to detect and quantify nearly 200 proteins and phosphoproteins across large number of TCGA tumor samples. The major limitation of the antibody based methods is the nonspecificity of the antibodies and low-throughput. Advancements of the tandem mass-spectrometry (LC-MS/MS) techniques at the level of MS resolution, accurate quantitation, and data analysis has made it a solid platform for simultaneous identification and quantification of the proteome of a cell [15]. The aim of quantitative proteomics is to obtain reliable quantitative information about all the proteins that fall within the mass-spectrometric dynamic range. In recent years the advent of cutting edge high-resolution “Orbitrap” mass-spectrometer instruments coupled with powerful computational platforms such as MaxQuant [16] facilitated the genome-wide identification and quantification of nearly all expressed proteins (roughly 18,000 proteins) in human cells and tissues which paved the foundation for the construction of the first draft of the human proteome [17, 18]. The application of mass-spectrometry based proteomics techniques has recently been extended to investigate the proteome alteration in various human cancer tissues [19]. However, unlike genomics and transcriptomics, mass-spectrometry (LC-MS/MS) based deep-proteomics techniques are under development to be routinely applicable in clinical settings. Nevertheless the promise this technology holds to identify novel diagnostic and prognostic biomarkers for cancer is enormous. Applications of mass-spectrometry-based OMICS techniques are summarized in Table 1.

The application of mass-spectrometric techniques is not limited to proteins and peptides but rather can be extended to small molecules such as metabolites. While proteomics covers the analysis of proteins, metabolomics on the other hand is characterized by the quantifications of metabolites that are synthesized as products of cellular metabolic activities, such as amino acids, fatty acids, carbohydrates, and lipids. The levels of metabolites and/or ratios of certain metabolites can be altered in disease states and thus reflect aberrant metabolic functions in complex diseases such as cancer [1, 20]. Metabolomics, though a relatively new field of OMICS, powered by the mass-spectrometry (LC-MS/MS) technology is beginning to provide biological insights into the changes of diverse metabolic pathways and fluxes in diseases states [21]. However, there are certain challenges (such as unknown metabolite identification, enormous diversity of metabolites and reproducibility) that must be overcome to materialize the full potential of mass-spectrometry-based metabolomics. The field of metabolomics is still emerging and embraces the potential to be highly effective in the discovery of biomarkers for cancer diagnosis and progression.

All the OMICS levels are important to decipher the complex phenotype of cells and organisms. Understanding the molecular basis of cellular phenotypes involving genes, RNA transcripts, proteins, and metabolites is particularly important because it not only gives an opportunity to predict the phenotypic alteration by examining the molecular signatures but also may serve as an unbiased platform to identify targets for therapeutic interventions. The next step towards the technological advances of OMICS fields would be to decrease sample processing/measurement time and increase reproducibility to firmly establish these techniques in clinical settings for diagnosis and prognosis of cancer.

## 2. Advantages of OMICS-Driven Studies in Cancer Context

Acquisition of cancer hallmarks allows the transition of a normal cell to malignancy. The hallmarks typically include complex phenotypic and molecular changes including uncontrolled and sustained proliferation, evading growth suppressors, resisting cell death, replicative immortality, angiogenesis, and metastasis [22]. Moreover mechanistic understanding of cancer progression though a series of experiments allowed us to get a glimpse of some other emerging hallmarks of cancer such as reprogramming of energy metabolism and evading immune destruction [22]. Attaining these hallmarks requires a series of alterations in the cellular machinery driven by molecular aberration in the genome, epigenome, transcriptome, proteome, and metabolome within cancer cells and/or tissues. For instance NGS of cancer cell genomes uncovered how activating mutations in certain proproliferative genes such as B-raf drives constitutive activation of mitogen-activated protein- (MAP-) kinase signalling which eventually manifests as uncontrolled proliferation of cells [23]. Molecular aberrations that drive the cancer are not restricted only to genomic mutational events but are extended into epigenome. For instance, silencing of certain tumor-suppressor genes can also be achieved through aberrant epigenetic mechanisms such as DNA methylation and histone modifications [24].

The hallmark—invasion and metastasis—requires the epithelial cells to undergo a transition towards mesenchymal phenotype thus enabling them to invade and migrate to distant sites for colonization. This complex phenotypic manifestation requires a complete gene-regulatory network that governs multiple genes/proteins to work in concert to achieve such dramatic changes. It has recently been shown that epithelial-mesenchymal transition (EMT) is indeed induced by certain transcription factors (TFs)—Snail, Slug, Twist, and Zeb1/2—coordinating the multistep process of invasion and metastasis [22, 25]. Transcriptomics techniques are suitable to uncover such TF-driven transcription regulatory networks that are assumed to be activated in a cancer context.

Although cell-fate decisions and phenotypic changes in cancer cells are initiated by transcriptional networks, these complex processes are executed by intracellular machineries composed of proteins. In this view, obtaining cancer hallmarks is essentially achieved by the alteration of the protein levels and/or PTMs (e.g., Phosphorylation status). For example, proproliferative signalling can be constitutively activated by upregulating the expression of the receptor proteins at the cancer cell surface [22] which can be detected by proteomics-centric studies. Unbiased global proteomics studies conducted by Tyanova et al. generated proteomic profiles comprising 19 proteins that can be successfully used to distinguish between oestrogen receptor positive (luminal), Her2 positive, and triple negative breast tumors [26].

The manifestation of cancer hallmarks does not leave the cellular metabolism unaffected. In a counterintuitive way, cancer cells are able to reprogram glucose metabolism and subsequent energy production by restricting oxidative phosphorylation even in the presence of oxygen. This phenomenon is commonly known as Warburg effect [22, 27]. In recent times with the technological progression of mass-spectrometry instruments, we can now better understand the metabolic reprogramming of cancer in great detail. For example, recently it has been shown that certain tumors are comprised of two metabolically distinct subpopulations of cells: one subpopulation has been shown to be glucose dependent and employ metabolic reprogramming to produce lactate as presumed in classical “Warburg effect”, whereas the second population channels the lactate from their neighbouring lactate producing cells as energy source for themselves [28].

All together it has now become apparent that to understand cancer progression, discover new therapeutic interventions, and develop novel cancer biomarkers we need to employ diverse OMICS strategies at multiple levels. While a single type of OMICS study can reveal a great deal of information at an unidirectional level (such as genomics can only reveal the mutational landscapes of cancer patients), the complexity of cancer-host interactions requires multidimensional approaches (such as genomics, epigenomics, transcriptomics, proteomics, and metabolomics) to portray the complete picture. Compared to single OMICS studies, multi-OMICS investigations have the potential to allow a deeper-understating of how the cancerous transformation is affecting the flow of information from one OMICS level to the next. Multi-OMICS approaches can bridge the link between cancerous genotype and the phenotypic characteristics.

## 3. Application of Multi-OMICS Approach: Success Stories So Far

Adaptation of cancer cells to a new cell-fate decision such as resisting apoptosis and phenotypic characteristics like metastatic invasion requires changes in the genome, epigenome, and gene expression profile that subsequently reshapes the proteome and metabolome to meet the challenges of altered cell-fate and phenotype. Integrating multi-OMICS profiles such as transcriptomics and proteomics offers the perfect strategy to unravel the information regarding differential abundance profile of mRNAs and proteins in varying conditions. In the following sections, we have discussed different integration approach of multi-OMICS data to understand the complexity of information processing systems in cancer cells.

### 3.1. Epigenomics versus Transcriptomics

The complexity of the mammalian cell is largely driven by the heritable genome constrained by epigenetic mechanism to regulate the expression of genes in different cellular contexts. This enables the cells to acquire the necessary functions for differentiation and proliferation. The epigenetic mechanisms are mediated through DNA/chromatin and histone protein modifications. In recent decades it has become apparent that the cancer epigenome harbors numerous alterations compared to their normal counterpart. For instance, genome-wide loss of methylation leading to aberrant unregulated expression of tissue specific and imprinted genes was observed to be associated with cancer [29, 30]. In line with this argument, studies have shown that hypomethylation in the promoter region of oncogenes,* RRAS, S100P*, and melanoma antigen family A1 (*MAGEA1*) activates their gene expression in gastric, pancreatic, and hepatocellular carcinoma, respectively [31]. In contrast to hypomethylation which was observed to manifest in global genome-wide manner, hypermethylation in different types of cancer occurs locally within specific segments of the genome. For instance promoter hypermethylation triggers the silencing of tumor-suppressor genes (TSGs),* BRCA1, CDKN2A*, and* MLH1*, thus making them unable to control cell cycle, apoptosis, and/or DNA repair [24, 32]. DNA-hypermethylation in CpG islands residing within promoter regions, known as CpG island methylator phenotype, has now turned out to be a tumor stratification strategy in many cancer types especially colorectal cancer [33]. Like the methylation pattern, many studies have now shown the association of altered histone modification profiles and cancer progression [30]. Aberrant epigenetic marks such as histone acetylation loss and altered H3K4, H3K9, and H3K27 methylation patterns are associated with various cancer types [30]. Since the manifestations of these epigenomic changes are essentially reflected in transcriptome level, integration of epigenomics and transcriptomics data have the potential to broaden our understanding of how molecular mechanisms initiate the acquisition of cancer hallmarks. Based on the casual relationship between methylation and gene expression it is generally accepted that hyper- and hypomethylation of promoter regions should essentially be reflected in decreased and increased expression of corresponding genes, respectively. Moreover histone methyl transferases gene,* EZH2*, was observed to be highly expressed in breast [34] and prostate cancer [35] implying bidirectional interactions between epigenome and transcriptome. Therefore in principle the reciprocal relationship between differential gene expression and epigenomic alterations can be investigated through the integration of ChIP-seq, methylomics, and RNA-seq data. Under this assumption a recent study conducted by Kelley et al. which integrated ChIP-seq and RNA-seq data obtained from patient-derived xenografts from head and neck squamous cell carcinoma (HNSCC) samples showed that H3K4me3 and H3K27ac histone marks are associated with tumor-specific transcriptional changes in their target genes including* EGFR, FGFR1, and FOXA1* [36]. Similarly another study by Bhasin et al. focused on the integration of genome-wide methylomics with publicly available RNA-seq data (obtained from TCGA) to characterize indolent and aggressive prostate cancer [37]. Here the authors identified certain differentially methylated regions (DMRs) within the promoter (e.g.,* CCDC8*) and gene-body (e.g.,* HOXC4*) of certain genes which showed strong negative and positive correlations, respectively with gene expression. These findings point towards a more complex scenario that a simple on- and off-state of genes is associated with the absence or presence of methylation. Methylation in the gene-body can also have a positive and direct correlation with gene expression [37]. A hypothesis involving the alternative splicing regulation by DNA methylation has recently been put forward to explain the correlation between gene-body methylation and gene expression [38]. A meta-analysis involving methylomes and gene expressions from 672 matched cancer and healthy tissues obtained from TCGA showed that hypermethylation in certain genomic regions is not necessarily linked to a decrease in expression of the corresponding genes [39]. This finding points towards the fact that genes may exhibit an unchanged expression even if their promoter region is methylated. New emerging hypotheses such as promoter cross-talk through a shared enhancer [40] and switching of promoter and enhancer domains [41] are proposed to suggest novel association mechanisms between genomic imprinting and gene expression for Nctc1 and Tet1/Tet2 genes, respectively. Whether this discordant relationship between methylation and gene expression is achieved by gene-specific or global mechanisms controlling gene expression bypassing the methylation status in cancer remains to be elucidated. Whatever the mechanisms underlying the discordance between epigenome and transcriptome are, these fundamental features of cancer cells can only be solved by harnessing the power of multi-OMICS technology.

### 3.2. Transcriptomics versus Proteomics

Over the last decade several large scale multi-OMICS studies involving transcriptomics and proteomics in mammalian cells demonstrated that the translational rate is the major contributor for the variation in protein abundance [18, 42, 43]. Earlier studies involving mass-spectrometry and microarray/mRNA sequencing (mRNA-seq) for the quantification of protein and mRNA levels of several thousand genes demonstrated the absence of a strong correlation between mRNA and protein levels. Rather mRNA and proteins levels showed moderate to poor correlation (coefficient of correlation R ≤ 0.4) [18, 42, 44]. This moderate correlation reflects that less than 40% variance on the protein levels is attributed to the mRNA levels. The remaining variance (>60%) is then essentially considered as the manifestation of differences in translational rate and protein degradation. In addition, using the information about degradation rates for mRNAs and proteins Schwanhäusser et al. estimated that transcription, mRNA degradation, translation, and protein degradation explains 34%, 6%, 55%, and 5% of protein abundance variation highlighting the role of translation as a dominant factor for regulating protein abundance [42, 44]. Schwanhäusser et al. showed that the translational rate can be considered as the most dominant factor governing the protein abundance. Although mRNA and protein levels may vary between cell types or tissues, the protein-to-mRNA ratio has been found to be highly conserved across twelve different human tissues for any given gene [18]. This conservation of the gene-specific protein-to-mRNA ratio showed the translational rate as an inherent and constant phenomenon for mRNA. Wilhelm et al. utilized this conserved protein-to-mRNA ratio for predicting the protein abundance for a gene in any given tissue from experimental mRNA abundance. Using the median protein-to-mRNA ratios per gene across twelve tissues, it was possible to predict protein levels from mRNA levels for every tissue. As a validation strategy they compared predicted protein abundance with experimental data to show strong correlation highlighting the role of translational rates defining the protein abundance [18]. However, it has not been investigated if the protein-to-mRNA ratios change or remain constant over time in highly evolving cells such as tumor cells. Therefore investigation of gene-specific protein-to-mRNA ratios in cancer cells in a temporal manner is necessary to uncover the dynamic interrelationship between transcriptome and proteome. The majority of the multi-OMICS studies directed towards deciphering the complex relation between transcriptome and proteome was performed in the context of steady state levels of proteins and mRNAs [17, 42, 44]. Studies of the transcriptome-proteome relationship under dynamic conditions are essential to understand how the information is propagated through these levels and ultimately contributes to the determination of cell-fate decisions. In order to dissect the individual roles of transcriptome and proteome in the context of dynamic cellular response, Jovanovic et al. showed that induction of novel cellular function in response to external stimuli is largely controlled by transcriptional alteration followed by proteome adaptations. In contrast the regulation of protein synthesis and degradation is mainly responsible for the maintenance of preexisting cellular functions [45]. However, with the cessation of the dynamic response, cells approach a new steady state. How cells maintain the newly acquired cellular function in a cancer context in the new steady state remains to be elucidated.

Transcriptome and proteome interrogations have been performed to decipher the aberrant molecular mechanisms in different cancer tissues such as oral squamous cell carcinoma [46], ovarian [47], breast [26, 48], colorectal [49], and lung [50] (Table 2). All these studies sought to investigate the tumor-specific transcriptome and proteome profiles to understand how the intricate molecular mechanisms drive the phenotypic changes in tumor cells. For instance, proteome profiling of breast tumors identified a set of 19 protein markers which could be used to stratify oestrogen receptor positive (luminal), Her2 positive, and triple negative breast tumors [26]. Out of the 19 markers analyzed, nine genes including MAPK3, MCM5, STMN1, and ENO1 exhibited concordant changes in protein and mRNA levels, rendering them as potential therapeutic targets of breast cancer [26]. Another study conducted by Li et al. analyzed genomics, transcriptomics, and proteomics of 33 samples, each comprising 11 non-small-cell lung carcinoma (NSCLC) tumor tissues, patient-matched tumor-free lung tissues, and patient-derived xenograft (PDX) [50]. By integrating the multi-OMICS data the authors argued that protein abundance is not a linear function of DNA copy number and mRNA abundance. Therefore mRNA and DNA copy number alteration (CNA) cannot serve as a proxy and good predictor for protein abundance. Intriguingly, they claimed this discordance of mRNA and protein levels to be highly gene-specific and consistent in both primary and PDX tumors [50].

In summary the integration of transcriptomics and proteomics data has already revealed some fundamental features of mammalian cellular systems. Although this multi-OMICS strategy is already in use to decipher molecular intricacy and mechanistic views of cancer pathophysiology, there are some fundamental questions which remain unanswered till date. For example, it is now accepted that mRNA and protein levels are not correlated in mammalian systems [17, 18, 42]. Whether this poor/moderate correlation is increased (or decreased) in a cancer context is unknown. Similarly whether a constant protein-to-mRNA ratio for a given gene within epithelial cells of different tissues changes upon cancerous transformation remains unresolved. We need to tackle these fundamental questions to be able to harness the full potential of the integration of multi-OMICS studies.

### 3.3. Proteogenomics: Connecting Proteome to Genome

While genomics, epigenomics, and transcriptomics studies have proved to be pivotal in gaining substantial insights into the architecture of the genome as well as the dynamics of transcriptome, the functional capacity of the genome that determines the cellular phenotype depends on the mechanistic power of the proteins. Moreover proteins are regulated extensively by PTMs and their interactions to other partner-proteins which cannot be predicted from genomics or transcriptomics data. To link genotype to phenotype, the “Clinical Proteomic Tumour Analysis Consortium (CPTAC)” has performed proteomic profiling of TCGA tumor specimens and linked to genomics, epigenomics, and transcriptomic profiles for colorectal (CRC) [49], breast [48], and ovarian [47] cancers (Table 2). Modest correlation between mRNAs and proteins was found for colorectal (0.47), breast (0.39), and ovarian (0.45) cancer as hypothesized by earlier studies. While the impact of copy number alterations (CNAs) was prominent on mRNA levels, a strong effect of CNAs was absent in protein levels as evident by the higher CNA-mRNA than CNA-protein correlations in CRC. Interestingly, amplification of chromosomal region 20q was associated with significant global changes in both mRNA and protein levels that are encoded by genes residing in these regions. These findings that emerged from the multi-OMICS data integration underscore the potential impact of 20q amplification in CRC, which was previously unknown. Hepatocyte-nuclear factor 4 alpha (HNF4A), Translocase of outer mitochondrial membrane (TOMM34), and SRC protooncogene, nonreceptor tyrosine kinase (SRC) proteins encoded by the 20q chromosomal region, were highly affected by the 20q amplification event and may play a vital role in attaining the cancer hallmark of sustained proliferation [49].

For breast cancer, when CNA-mRNA and CNA-protein pairs were analyzed for 478 oncogenes and TSGs, these cancer-related genes where found to be enriched in the subset that exhibited concordance on both CNA-mRNA and CNA-protein levels compared to the subset that only correlates on CNA-mRNA but not on CNA-protein levels [48]. This finding is of particular importance because it underscored that the tumor-promoting CNA events are more likely to have an effect on both the protein and mRNA levels. In contrast nontumorigenesis CNA events are more likely to lose their impact and may be neutralized on the protein level rather than on the mRNA level. In addition the proteogenomic approach has proved to be particularly effective in identifying possible druggable targets. Proteogenomic analysis of breast cancer tissues resulted in the identification of such candidate proteins,- CDK12, TLK2, PAK1, and RIPK2, that showed gene-amplification-driven proteogenomic patterns [48].

**Figure 3:**
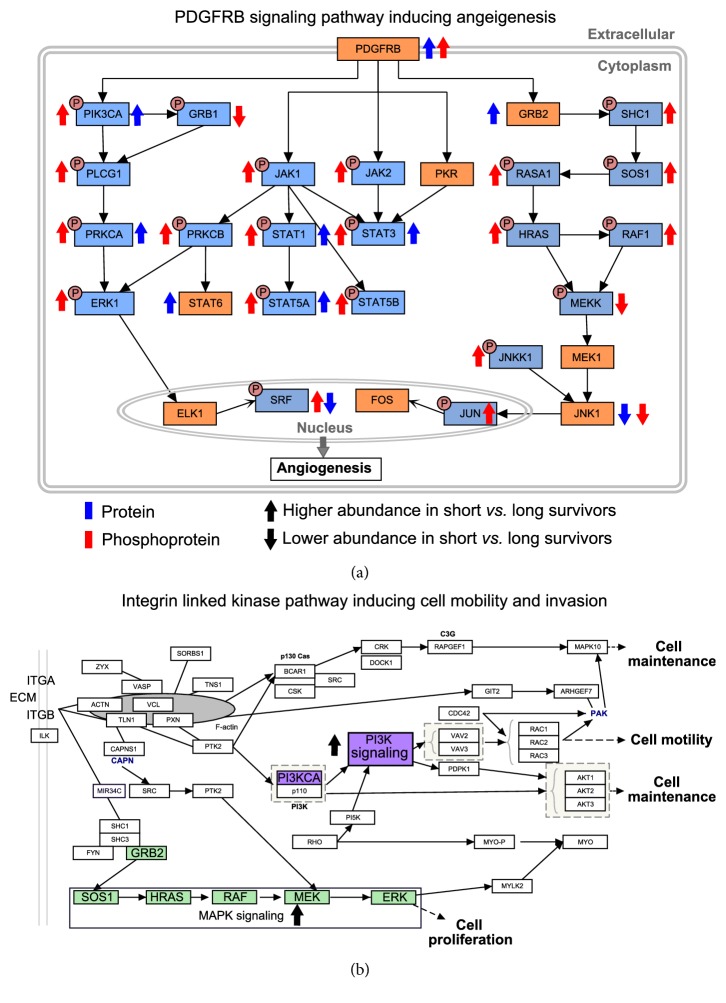
**Proteomics and phosphoproteomics driven identification of the aberrant regulation of signalling pathways leading to poor patient survival.** (a) Aberrant PDGFRB signalling pathway induces angiogenesis in patients and results in poor survival. Phosphorylated and unphosphorylated forms of proteins are indicated by blue and orange color. The directions of arrows indicate the regulation—up and down arrows indicate upregulation and downregulation, respectively. Colors of the arrows indicate the phosphoform or the total protein detected by (phospho)proteomics experiments. Blue and red arrows indicate phospho- and total protein, respectively. (b) Integrin-linked kinase pathway induces cell mobility and invasion, leading to poor patient survival. Two signalling pathways, MAPK (green) and PI3K (purple), are highlighted. Both these pathways were found to be upregulated in cancer patients with poor survival.

High-Grade Serous Ovarian Cancer (HGSC) is characterized by high CNAs leading to chromosomal instability (CIN) [47]. CNAs may have an impact on the abundance of mRNA/proteins in the same (cis-effect) and/or different (trans-effect) locus. In colorectal cancer CNA-driven trans-effects were observed on both mRNA and protein levels [49]. On the contrary, in ovarian cancer, trans-effect of CNAs on protein abundances was independent of change in mRNA levels. For instance, CNA on specific segments on Chromosome 2 was observed to have strong trans-effect on more than 200 proteins whereas such effects on mRNA levels were very small [47]. As a plausible mechanism to explain the trans-effect of CNA on protein levels without affecting the corresponding mRNA levels, the authors argued that cis-regulation of RNA-binding proteins or microRNAs that are associated with mRNA stability and translational process may be responsible for such trans-effect on protein levels. The proteins for which the abundance is modulated by CNAs mostly belong to cell invasion and migration indicating a possible role of CNA-driven proteogenomic events in attaining these hallmarks of cancer [47]. Next, correlation analysis between CIN and protein abundances led to the identification of two candidate proteins CHD4 and CHD5 that are normally associated with chromatin organization. Abundance variation of these proteins can potentially elicit CIN in ovarian cancer [47]. Under the assumption that PTMs such as phosphorylation may also play a crucial role in activating the signalling cascade to attain cancer hallmarks, proteomic and phosphoproteomic data was integrated with transcriptomic data for ovarian cancer. This integration approach was particularly helpful in the identification of the differentially regulated pathways, PDGFR-beta signalling pathway associated with angiogenesis and integrin-linked kinase pathways associated with cell mobility and invasion (Figure 3), that may serve as a predictor of patient survival. A recent study carried out by the TCGA consortium used integrated genomics, transcriptomic, epigenomics, and proteomics approaches to identify distinct molecular subtypes of the Testicular Germ Cell Tumors (TGCT) [51].

Overall the proteogenomic approach underscores the complementarities of proteomics/phosphoproteomics dataset to harmonize genomics/epigenomics and transcriptomics to gain deeper understanding into the molecular mechanisms that help malignant cell to attain cancer hallmarks. These studies also corroborated the previous notion that mRNA levels are not a good proxy for protein abundance and thus cannot be predicted only from mRNA data. Moreover CNAs driven changes in protein abundance may serve as reliable marker for cancer prognosis and treatment stratification. Taking account of all the insights gained from these three proteogenomic studies, it can be safely assumed that the integration of multi-OMICS data may have significant impact on the diagnosis, prognosis, and treatment-outcome of individual cancer patients in a personalized manner.

### 3.4. Transcriptomics versus Metabolomics

Unbiased metabolomic-profiling of cancer cell is becoming increasingly popular due to its potential to identify and quantify novel oncometabolites which may serve as biomarkers for different cancer types. Apart from obvious advantages in identifying novel cancer biomarkers, metabolomics may provide key-insights into the pathophysiology of cancer when merged with other OMICS data. In order to extract biologically meaningful insights from metabolomics data and contextualize the differential abundances of oncometabolites, multi-OMICS data integration is necessary. In the following examples, we have shown how metabolomics data integration to other OMICS can be used not only to advance our understanding into the molecular mechanism of cancer progression but also to predict the survival rates of cancer patients.

In a multi-OMICS integration study, Terunuma et al. showed that levels of the oncometabolite-2-hydroxyglutarate (2HG) were elevated in predominantly ER-negative subgroup of breast tumors and associated with poor clinical outcome. Moreover, integration of metabolomics with genome-wide methylomics data revealed that the subtype of breast tumors marked by elevated 2HG levels exhibited a hypermethylation phenotype [52]. Corroborating with this result, earlier studies also demonstrated the association between the elevated 2HG levels and DNA-hypermethylation and enhanced histone methylation causing epigenetic alterations in gliomas [53] and leukemias [54].

In another study, integration of publicly available transcriptomic and metabolomic datasets showed a strong enzyme-metabolite concordance in breast cancer and hepatocellular carcinoma tissues [55]. Both breast and hepatocellular cancer exhibited increased gene-metabolites associations in comparison to adjacent noncancerous tissues. The authors argued that alerted gene-regulatory networks in cancer context may force the changes in cancer-related metabolic pathways causing an abundance change in the metabolite levels [55]. Based on the multi-OMICS data integration, a prediction model, developed and validated against a large cohort of breast cancer patients, showed several cancer-related metabolites; namely, glucose, Glycine, serine, and acetate are significantly associated with patient survival [55]. A similar OMICS integration approach including metabolomics and transcriptomics was applied to identify potential diagnostic and prognostic cancer biomarkers for prostate [56] and cervical cancers [57]. Ren et al. identified the accumulation of certain metabolites such as S-adenosylhomoserine (SAH), 5-methylthioadensine (MTA), and S-adenosylmethionine (SAM) in prostate cancer compared to noncancerous tissues [56]. Elevated expression of Glycine N-methyltransferase (*GNMT*) evident from transcriptomics analysis was assumed to be responsible for the induction of SAH and was proposed as a tumor-susceptibility gene in prostate cancer [56]. On the other hand, Yang et al. identified five metabolites, bilirubin, LysoPC(17:0), n-oleoyl threonine, 12-hydroxydodecanoic acid, and tetracosahexaenoic acid, as candidate biomarkers for cervical cancers [57]. Integration strategy leads to the reconstruction of an interconnected gene-metabolic network where seven biochemical pathways were used to identify five candidate metabolite biomarkers [57]. The metabolomics integration studies provided systems-level insights into altered metabolic networks that are tightly regulated with transcriptional network. These interconnected networks could potentially serve as a platform for the identification of novel therapeutic targets and biomarkers for cancer.

**Figure 4:**
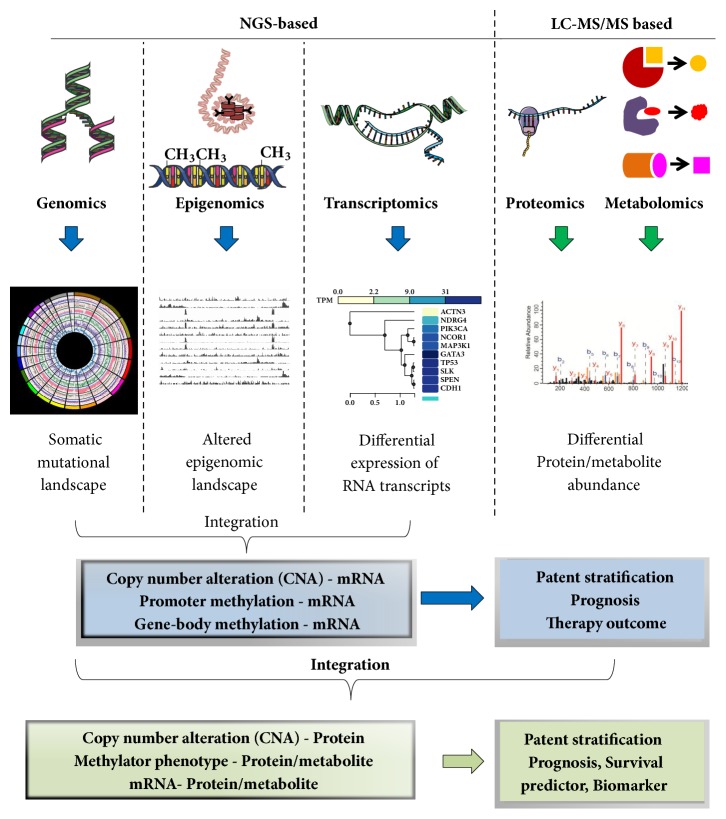
**Application of NGS and mass-spectrometry (LC-MS/MS) based OMICS techniques in cancer research.** Genomics, epigenomics, and transcriptomics are based on NGS techniques; whereas proteomics and metabolomics are driven by mass-spectrometric (LC-MS/MS) technique. The principal application of genomics, epigenomics, and transcriptomics is screening of genome-wide somatic mutations, identification of altered epigenomic landscape, and exploring differential RNA expression, respectively. The major application of proteomics/metabolomics is identification of differentially regulated proteins/phosphoproteins/metabolites. The integration of NGS-based techniques can identify the concordance or discordance between copy number alterations (CNAs), promoter/gene-body methylation, and RNA levels. Integration of NGS and LC-MS/MS based techniques may result in the correlation analysis between CNAs, promoter/gene-body methylation, and mRNA levels with protein/metabolite levels.

In the era of cutting-edge OMICS technologies, the multi-OMICS integration approaches have emerged as a powerful strategy to better understand the molecular basis of cancer and eventually to develop intervention strategies through the identification of robust patient stratification methods, biomarker for early cancer diagnosis/prognosis, and prediction of therapy-outcome.  Figure 4 represents the different methods of multi-OMICS data integration and their subsequent application in cancer research.

## 4. TCGA as a Resource for Multi-OMICS Platform

With the goal of creating a publicly available comprehensive “atlas” of the molecular alterations in the cancer cells, The Cancer Genome Atlas (TCGA), so far, has performed integrative analysis of more than 30 human tumor types [13]. The TCGA Research Network is engaged in cataloguing aberrations in the DNA and chromatin of the cancer-genomes from thousands of tumors by matching with the normal genomes and linking these aberrations to RNA and proteins levels. The main TCGA-strategy of multi-OMICS integration involves genomics, epigenomics, and transcriptomics which has been successfully implemented in the investigation of various cancer types including testicular germ cell tumors [51], soft tissue sarcomas [58], gastrointestinal adenocarcinomas [59], clear cell renal cell carcinoma [60], prostate [61], urothelial bladder carcinoma [62], gastric adenocarcinoma [63], oesophageal carcinoma [64], acute myeloid leukemia [65], melanoma [66], and lung adenocarcinoma [67] (Table 2). For colorectal [49], breast [48], and ovarian [48] cancer, mass-spectrometry-based proteomics data has been integrated into the existing strategy of OMICS integration as described earlier (see Table 2). For glioblastoma [68] RPPA based targeted proteomics was integrated to existing strategy of OMICS data integration.

The new TCGA-atlas called the “Pan-Cancer initiative” has been developed and is dedicated to comparing and contrasting among the genomic, epigenomic, and transcriptomic alterations found in numerous tumor types [13]. The pan-cancer analysis involving multi-OMICS data in combination with robust bioinformatics methods and statistical tools offers a unique platform to identify common molecular signatures for the stratification of patients with different cancer types and uncover shared molecular pathology of different cancer types for designing targeted therapies. With the genomics, epigenomics, and transcriptomics data from over 11,000 tumors representing 33 of the most prevalent forms of cancer, the Pan-Cancer Atlas presents the unique opportunity for comprehensive and integrated analysis to broaden our current understanding of how, where, and why a normal cell attains cancer hallmarks. Analysis of the enormous amount of cancer-specific data deposited in TCGA requires special bioinformatics methods and techniques to be able to extract biologically meaningful information. Various data analysis and visualization platforms have been developed to assist the rapid analysis of TCGA data. For instance, cBioPortal originally developed at Memorial Sloan Kettering Cancer Center provides opportunities like visualization, analysis, and download of large scale cancer genomics data sets [69].

## 5. Future of Multi-OMICS Studies: Emerging Era of Systems Biology

Recent advances in high-throughput NGS and mass-spectrometric techniques enabled a paradigm shift from studies involving discrete biochemical reactions and signalling pathways to large scale studies attempting to analyze the whole cellular system. With powerful computational tools one can identify the link between genomic aberrations with differentially expressed mRNAs, proteins, and metabolites that are associated with a cancer-driven cellular perturbation. Integration of multi-OMICS data provides a platform to link the genomic/epigenomic alterations to interconnected transcriptome, proteome, and metabolome networks, which underlie the cellular response to a perturbation. This vision provides an opportunity to better understand cellular response on the systems level but poses a challenge for systems biology driven modelling at the same time. The next phase of systems biology research will focus on models that can deal with thousands of mRNA, protein, and metabolite changes in a dynamic manner. Systems biology approach has the potential to develop effective strategies to administer personalized cancer therapy [70]. The aim of the systems biology approach is to develop predictive models that are refined and constrained by experimental validations. These predictive models will be particularly beneficial to select patients based on personalized multi-OMICS data and stratifying the patients to determine who are most likely to benefit from targeted therapies [71]. In summary systems biology models driven by multi-OMICS data may help to increase the onco-drug efficacy and overcome the chemo-/immunotherapy resistance phenotype of cancer cells rendering them vulnerable to targeted therapies and ultimately in improving the quality of life of patients.

## Figures and Tables

**Table 1:** Different omics techniques and their applications.

**Omics**	**Type**	**Principle**	**Throughput**	**Application **
Genomics	Whole exome sequencing	NGS	high	Genome-wide mutational/ analysis

	Whole genome sequencing	NGS	high	Exome-wide mutational analysis

	Targeted gene/exome sequencing	Sanger-sequencing	Low	Mutational analysis in targeted gene/exon

Epigenomics	Methylomics	Whole-genome bisulfite sequencing	High	Genome-wide mapping of DNA methylation pattern
	ChIP-sequencing	Chromatin IP∗ and NGS	high	Genome-wide mapping of epigenetic marks

Transcriptomics	RNA-sequencing	NGS	High	Genome-wide differential gene expression analysis
	microarray	Hybridization	High	Differential gene expression analysis

Proteomics	RPPA	Antibody based	Low	Differential protein abundance analysis
	Deep-proteomics	Mass-spectrometry	high	Genome-wide differential protein expression analysis

Metabolomics	Deep-metabolomics	Mass-spectrometry	high	Differential metabolite expression analysis

**Table 2:** Multiomics studies focusing on cancer.

**PMID**	**Tumor type**	**Cohort**	**Samples no#**	**Genomics**	**Methylomics**	**Transcriptomics**	**Proteomics**	**Metabolomics**
28878238	OSCC^1^	Taiwanese	T=38, N=38	+		+	+	

27372738	Ovarian	TCGA^2^	T=174	+	+	+	+	

27251275	Breast	TCGA^2^	T=105	+	+	+	+	

25043054	Colorectal	TCGA^2^	T=96	+	+	+	+	

26725330	Breast	N/A	T=40			+	+	

25429762	Lung	N/A	T=11, N=11	+		+	+	

28947419	Head/Neck Tumor	N/A	T=47			+		

26628371	Prostate	N/A	Unknown		+	+		

28225065	Cervical	N/A	T=52			+		+

26545398	Prostate	N/A	T=25, N=25			+		+

27406679	Breast and HCC^3^	N/A	N^3^=105			+		+

24316975	Breast	N/A	T=67, N=65		+	+	+	+

29898407	TGCT^4^	TCGA^2^	T=137	+	+	+	+	

29100075	Soft Tissue Sarcomas	TCGA^2^	T=206	+	+	+		

29622466	GIAC^5^	TCGA^2^	T=921	+	+	+		

29925010	ccRCC^6^	TCGA^2^	T=400	+	+	+		

26544944	Prostate	TCGA^2^	T=333	+	+	+		

24476821	UBC^7^	TCGA^2^	T=131	+	+	+		

26091043	Melanoma	TCGA^2^	T=331	+	+	+		

25079317	GA^8^	TCGA^2^	T=295	+	+	+		

28052061	OEC^9^	TCGA^2^	T=164	+	+	+		

24120142	Glioblastoma	TCGA^2^	T=500	+	+	+	+	

23634996	AML^10^	TCGA^2^	T=200	+	+	+		

25079552	LUAD^11^	TCGA^2^	T=230	+	+	+		

T: Tumor, ^1^OSCC: Oral Squamous Cell Carcinoma, ^2^TCGA: The Cancer Genome Atlas, N: Normal, ^3^HCC: Hepatocellular Carcinoma, ^4^TGCT: Testicular Germ Cell Tumors, ^5^GIAC: Gastrointestinal Adenocarcinomas, ^6^ccRCC: Clear Cell Renal Cell Carcinoma, ^7^UBC: Urothelial Bladder Carcinoma, ^8^GA: Gastric Adenocarcinoma, ^9^OEC: Oesophageal Carcinoma, ^10^AML: Acute Myeloid Leukemia, ^11^LUAD: Lung Adenocarcinoma.
